# Optimizing Winter Air Quality in Pig-Fattening Houses: A Plasma Deodorization Approach

**DOI:** 10.3390/s24020324

**Published:** 2024-01-05

**Authors:** Liping Zhang, Meng Zhang, Qianfeng Yu, Shiguang Su, Yan Wang, Yu Fang, Wei Dong

**Affiliations:** 1 Agricultural Economy and Information Research Institute, Anhui Academy of Agricultural Sciences, Hefei 230001, China; sangold@163.com (L.Z.); zhangmengchn@163.com (M.Z.);; 2 School of Mechanical and Electronic Engineering, Suzhou University, Suzhou 234000, China; 3 Animal Husbandry and Veterinary Research Institute, Anhui Academy of Agricultural Sciences, Hefei 230001, China

**Keywords:** fattening house, low-temperature plasma, odor removal device, ventilation system, air quality

## Abstract

This study aimed to evaluate the effect of two circulation modes of a plasma deodorization unit on the air environment of pig-fattening houses in winter. Two pig-fattening houses were selected, one of which was installed with a plasma deodorizing device with two modes of operation, alternating internal and external circulation on a day-by-day basis. The other house did not have any form of treatment and was used as the control house. Upon installing the system, this study revealed that in the internal circulation mode, indoor temperature and humidity were sustained at elevated levels, with the NH_3_ and H_2_S concentrations decreasing by 63.87% and 100%, respectively, in comparison to the control house. Conversely, in the external circulation mode, the indoor temperature and humidity remained subdued, accompanied by a 16.43% reduction in CO_2_ concentration. The adept interchange between these two operational modes facilitates the regulation of indoor air quality within a secure environment. This not only effectively diminishes deleterious gases in the pig-fattening house but also achieves the remote automation of environmental monitoring and hazardous gas management; thereby, it mitigates the likelihood of diseases and minimizes breeding risks.

## 1. Introduction

Pig farming in China is a traditional industry that has experienced long-term rapid growth in pig production. Currently, large-scale breeding has emerged as the dominant trend in the pig-breeding industry, leading small- and medium-sized free-range farmers to gradually withdraw from the market. Therefore, the market is now filled with large-scale, standardized, and ecologically conscious breeding enterprises. While large-scale pig farming has advantages such as standardization, high efficiency, low costs, and high output, it also poses a series of challenges such as the high number of pigs, high breeding density, limited activity range, and multiple environmental stress factors. The suboptimal breeding environment of pig farms results in poor pig health, which creates favorable conditions for the occurrence and spread of diseases [1]. Under the large-scale production mode of pig farms, the increasing feeding density, harmful concentration of gases, dust content, and microbial content in the air all have a significant impact on the development and production performance of pig farms.

Monitoring and controlling the air quality on pig farms has become a pivotal use of technology in pig farming [2,3]. Air quality has a direct impact on the growth processes and production performance of pigs, which in turn affects the physical health of farm workers, the pork quality, and the overall economic benefits of the farm [4]. Additionally, waste discharged from pig farms can pollute the surrounding ecological environment. Toxic and harmful gases inside pig houses, such as NH_3_, H_2_S, and CO_2_, are primarily produced through the daily respiration and excretion of feces and urine from the pig herd, as well as through the decomposition of the organic substances used in pigsty bedding [5]. Germs can also be transmitted through the air, causing large-scale infections. While, traditional disinfection techniques for air-borne germs and inside premises, such as water washing, liquid cleaning, activated carbon adsorption, and primary biological deodorization, tackle the problem from various perspectives [6,7]; however, they all suffer from drawbacks such as low efficiency and high work intensity.

Prevalent strategies for the treatment of exhaust gases in intensive livestock houses predominantly revolve around reduction measures focused on end-of-pipe emissions [7]. The composite spray method, encompassing acid washing and water washing, stands out as the most widely employed technique [8]. This method demonstrates versatility in purifying both particulate matter and gaseous pollutants, rendering it highly adaptable to exhaust gas purification in livestock houses. Additionally, mechanical ventilation serves as a common measure on pig farms, effectively concentrating and organizing gas emissions within the confines of the houses [9]. This organizational approach facilitates the potential treatment of emitted odors beyond the perimeters of the houses. However, the sustained emissions of high volumes of air with low concentrations poses technical challenges to the purification process. The challenges are particularly pronounced in economically disadvantaged areas, where a significant number of farmers opt for natural ventilation as the sole method for indoor air purification.

In recent years, numerous scholars have conducted extensive research on the application of plasma technology, including material surface modification [10], waste treatment [11], medical disinfection and sterilization [12], fresh air system purification [13], and other fields. Plasma technology can be divided into high-temperature plasma and low-temperature plasma [14]. Low-temperature plasma, with its unique technological advantages such as low gas temperature, strong chemical activity, and lack of pollution [15], is more suitable for medical, agricultural, and other fields. It will not cause damage to biological factors and the environment, making it an ideal option.

Plasma air disinfection and sterilization technology can customize the air disinfection and sterilization system according to the specific working conditions of breeding enterprises, so as to realize the air purification and disinfection of breeding locations, such as pig-fattening houses. Low-temperature plasma can eliminate odors and effectively decompose and treat pollutants through high-energy electrons and free radicals [16,17], thereby degrading those pollutants. On the one hand, it can purify the air, and on the other hand, it can achieve the continuous inactivation of viruses in the air [18], blocking their passage through the air. This can greatly reduce the probability of plague occurrence or other airborne infections, ensure the safety of livestock and poultry, avoid property damage, and contribute to maintaining public health.

Inaugurated in 1984 for the treatment of volatile organic compounds emanating from military waste, low-temperature plasma technology has since undergone research into various applications [19]. Zhu conducted a comprehensive study on the impact of the diverse process parameters of low-temperature plasma technology in the removal of H_2_S gas [20]. Xie, in turn, employed varying degrees of plasma to address NH_3_ and H_2_S, culminating in findings that underscore the considerable promise of plasma in mitigating these gases [21]. Moreover, the fusion of low-temperature plasma with catalyst oxidation has been investigated to synergistically reduce the concentration of harmful gases [22]. Xu’s research, for instance, revealed that utilizing CuO/AC as a catalyst significantly enhances the removal efficiency of benzene, with an increasing trend noted as the CuO content increases [23]. Notably, documented instances exist where plasma technology has been harnessed for industrial purposes, specifically in purifying the air within livestock facilities. Saoud, for instance, integrated photocatalysis and plasma to diminish NH_3_ and Pro-pionaldehyde in livestock enclosures, attaining degradation efficiencies of 72% and 83%, respectively [24]. Nevertheless, these studies still position the plasma generator directly within the confines of the livestock houses, leading to the entire purification process occurring indoors and consequently yielding suboptimal controllability.

In this article, a deodorization device architecture is proposed, which combines barrier dielectric discharge and microwave photolysis technology. Its feasibility is verified through practical application in pig pens, achieving remotely operable 24-h air quality monitoring and purification in those pig pens. Automated systems can provide real-time monitoring and management of environmental conditions. This allows for immediate responses to any deviations from optimal conditions, minimizing the risk of stress or health issues in pigs.

## 2. Materials and Methods

### 2.1. Experimental Rooms

The experimental site was situated within a pig farm in Anhui Province, China, at coordinates 116^◦^ 28′ E, 33^◦^ 22′ N. Two pig-fattening houses, each with an area of roughly 300 square meters and a stock of 200 fattening pigs (large white × long white × Duroc), were selected for the experiment. The batch of pigs entered the houses on 12 October 2022 and exited on 7 February 2023, with a weight of 45 ± 3 kg at the time of entry. The experiment lasted for 60 days from 1 November 2022 to 30 December 2022. House 1 was furnished with a plasma deodorizer and ventilation system, and served as the experimental house. Due to notable diurnal variations in pig behavior, coupled with the movement of staff members entering and exiting the premises for feeding or cleaning purposes, significant fluctuations existed in diverse monitoring parameters. Consequently, the experimental timeline was selected of the period covering 19:00 p.m. to 7:00 a.m. of the following day. House 2, which served as the control house, was ventilated by opening windows at a fixed time every day. Additionally, to ensure that the two working modes in House 1 did not interfere with each other, the equipment was non-operational except during testing. Throughout the experiment, normal feeding and hygiene protocols were maintained in both pig houses, with four stages of daily feeding and one stage of fecal cleaning.

### 2.2. Measurements

By utilizing the Internet of Things and big data technology, remote monitoring and measurement of the environmental indicators in pig houses are now feasible [25]. The integration of wireless modules, sampling devices, sensor detection modules, and video transmission modules allows for the real-time collection and transmission of environmental quality indicators in pig houses to the central computer, enabling long-term monitoring to procure continuous data. To obtain air quality indicators, a monitoring system should be installed at the central positions of each of the two pig houses [26]. Users can remotely access real-time temperature and humidity data, as well as data on the concentrations of NH_3_, H_2_S, CO_2_, and O_3_ gases in the two pig houses through web pages. All sensors automatically gathered and recorded data hourly between 20:00 p.m. and 7:00 a.m. every day, with each indicator being recorded 12 times per day. The internal circulation experimental data for House 1 were obtained by averaging the values of odd days, while the external circulation experimental data were obtained by averaging the values of even days. Meanwhile, the control data for House 2 were obtained by averaging the values every 2 days. While conducting the data analysis, we identified individual data points with notable deviations. We attributed this to the harsh environment inside the pig houses, potentially affecting the normal operation of the data sensors. Consequently, alongside regular sensor inspections, we have excluded data exhibiting significant errors.

### 2.3. Ventilation System

In general, the air quality within a pigsty tends to be inferior to that of the external environment, and the implementation of ventilation serves as a mitigating measure to neutralize indoor pollutants. The principal objective of ventilation in swine housing is to adjust the ambient temperature and humidity, nullify the unpleasant indoor gases, expel surplus carbon dioxide, and enhance the quality of the indoor air [27]. In House 2, the most commonly used method of ventilation, which dates back to early pigsties, was opening windows without any supplementary means of ventilation [28]. House 1 was segregated into two modes: external circulation mode (EC-mode) and internal circulation mode (IC-mode), with different ventilation methodologies. Users possessed the capability to transition between modes using either the external control panel of the apparatus or a dedicated mobile application.

**Figure 1:**
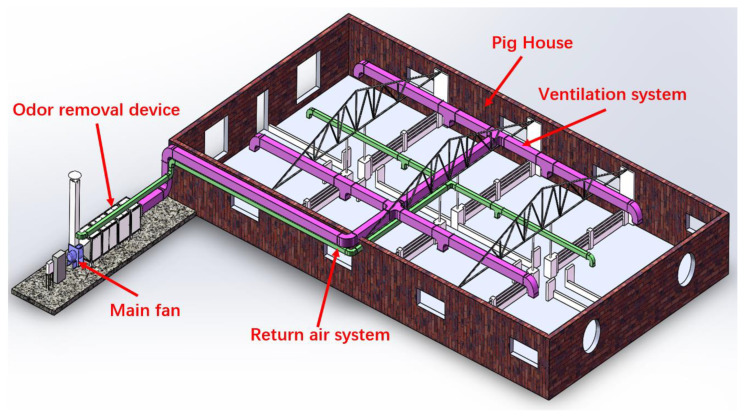
Schematic diagram of the ventilation system.

As shown in Figure 1, the experimental structure was equipped with a ventilation system, comprising a primary fan, outlet duct, return duct, and exhaust chimney, to assist in the operation of the deodorizing device. House 1 was subdivided into eight sections. Considering that noxious gases within the pig house primarily stem from the life processes of pigs, a measure was implemented to uniformly disperse these detrimental gases within the premises. Consequently, 200 fattening pigs were apportioned to each designated area, comprising 25 pigs per sector. There was an air inlet located directly above the middle of each section, and a total of four return air outlets were positioned above the corridor. The unclean air within the house infiltrated the deodorizing device through the air inlet and after processing was reintroduced into the house (IC-mode) or expelled into the atmosphere (EC-mode).

#### 2.3.1. External Circulation System

Upon activation of the external circulation mode, the windows throughout the room were opened, while the valves in the device were directed towards the return air duct and the exhaust chimney was shut. A fan was utilized to extract the polluted air from inside the house, which was then treated and expelled from the chimney into the atmosphere. As shown in Figure 1, during fan operation, indoor polluted gas was conveyed from the violet conduit to the odor removal device. Following treatment, it was released into the atmosphere via a chimney. This created a pressure differential between the interior and exterior of the dwelling, allowing for the influx of fresh air from outside and achieving a complete exchange of air.

#### 2.3.2. Internal Circulation System

When the internal circulation mode was activated, all windows in the room were sealed shut, while the valves in the device were directed towards the exhaust chimney and the return air duct was closed. The air outlet and return mechanisms inside the house were powered by fans, which sucked in the contaminated air through the outlet duct and delivered it to the deodorization device for processing. As depicted in Figure 1, similar to the external circulation, indoor polluted gas was transported to the odor removal device through violet color conduits, with the treated gas subsequently reintroduced indoors through green color conduits. Once the air had been purified, it was then returned to the house through the return duct, thereby achieving a closed-loop circulation system.

#### 2.3.3. Ventilation Quantity

**Figure 2:**
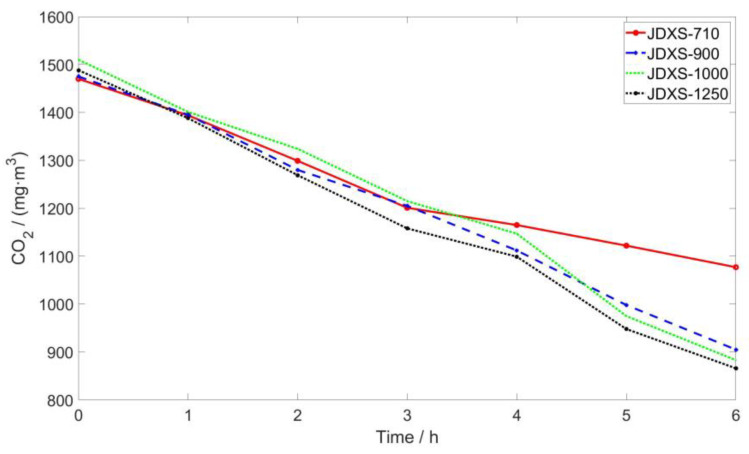
Trend in the CO_2_ concentration during the operation of different fans.

Throughout the purification process, indoor pollutants traversed the ventilation system to undergo purification in the deodorization device. The efficacy of the deodorization device was contingent upon the proficiency of the ventilation system. In our preliminary experiment, we assessed the impact of various fan power levels on the system, as shown in Table 1. Given the experiment’s objective to juxtapose the effects of diverse ventilation rates on indoor air circulation efficiency [29], the CO_2_ concentration in the EC-mode served as the reference indicator. For each experiment, the indoor CO_2_ concentration was maintained at approximately 1500 mg/m^3^, and the changes in CO_2_ over a 6-h period are delineated in Figure 2. The JDXS-900 demonstrates notably superior efficiency in reducing the CO_2_ concentration within 6 h compared to the JDXS-710, while the advantages of the JDXS-1000 and JDXS-1250 are inconspicuous, signifying that the JDXS-900 adequately meets the requisite standards.

### 2.4. Odor Removal Device

**Figure 3:**
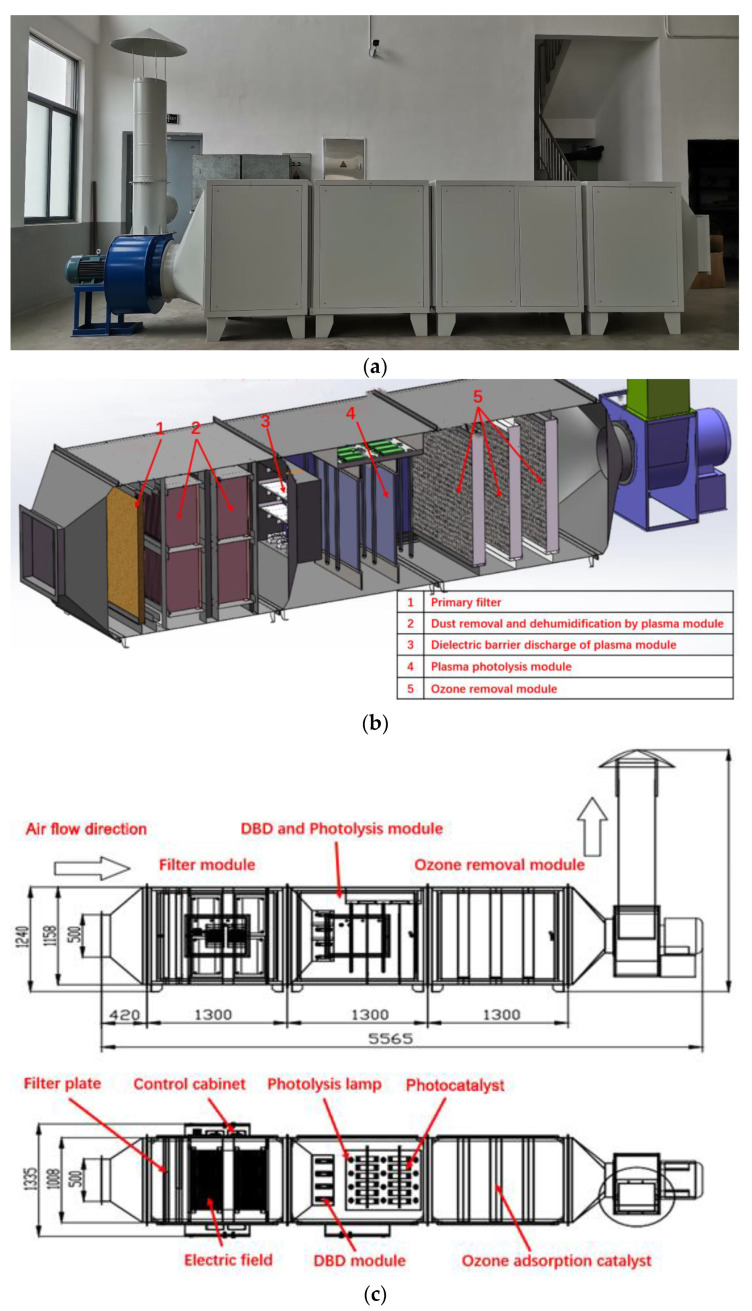
Schematic diagram of the plasma system: (**a**) physical image; (**b**) sectional drawing; (**c**) structural diagram.

The odor removal device comprises four essential components: a primary filter plate (which includes a dehumidification fan and a dust removal electric field), a dielectric barrier discharge plasma generator, a microwave photolysis module, and an ozone removal module, as shown in Figure 3 and Table 2. The total power consumption of the equipment, including the main fan, amounts to 7.9 kW. The device was located on the exterior side of experimental House 1.

#### 2.4.1. Primary Filter Plate

The primary filter plate is composed of a dehumidification fan and a dust removal electric field, and it serves as the first filtration stage before plasma odor removal. Its main function is to remove water and various dust particles larger than 5μm in the air [30]. This step is essential in preventing the adhesion of large particles to the discharge electrode, avoiding any potential sparks between electrodes, and ensuring the stable operation of the plasma generator [31]. The dehumidification fan enhances the air flow, facilitates water evaporation, and minimizes the possibility of interfering with the normal functioning of the equipment. The dust removal electric field charges particles, and the charged particles move to the dust collection electrode plate in the dust collection area.

#### 2.4.2. Dielectric Barrier Discharge Module

**Figure 4:**
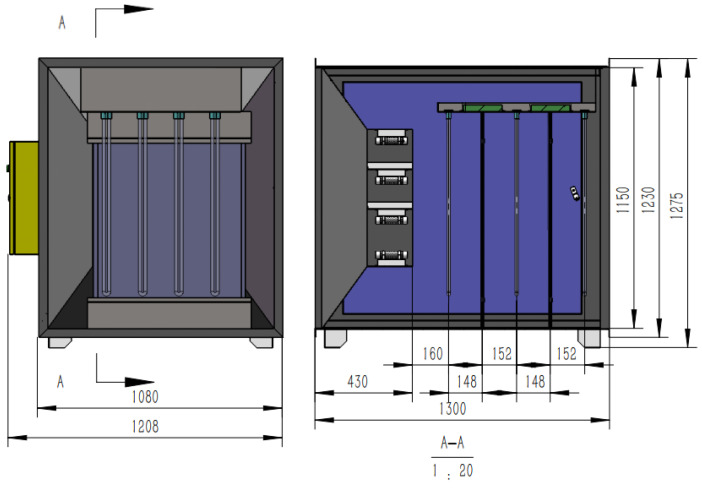
Dielectric barrier discharge module.

Dielectric barrier discharge (DBD) plasma integrates ozone oxidation, free radical oxidation, pyrolysis, and high-energy electron radiation [32,33,34], making it highly effective in eliminating various gaseous pollutants, particularly H_2_S and NH_3_ [35], which are otherwise challenging to degrade. This treatment technique offers several advantages, including a broad treatment range, high treatment efficiency, and no secondary pollution [36,37]. The process of treating harmful gases using DBD plasma involves placing a barrier medium between the electrodes to separate them [38]. The structure of the DBD module is shown in Figure 4, under the influence of high voltage, the gas between the discharge electrodes is broken down, creating a non-uniform spark channel. The ionized electrons move rapidly towards the anode under the influence of the electric field, further ionizing the discharge gas, resulting in the generation of an electron flow that gradually fills the entire discharge channel. Consequently, a uniform DBD is formed between the two electrodes, generating highly active particles [39] that facilitate the decomposition and oxidation–reduction reactions with pollutants in the gas. Additionally, the heat generated by the discharge can also cause a certain degree of pyrolysis of the pollutants.

#### 2.4.3. Microwave Photolysis Module

Microwave photolysis utilizes a microwave ultraviolet lamp to generate multi-band, high-strength ultraviolet light [40], which decomposes oxygen molecules in the air to produce O_3_, increases the motion speed of ionized molecules, enhances the impact energy of photons, and achieves the effect of cracking pollutants. Through the joint action of microwave, ultraviolet, O_3_, and hydroxyl radicals, the pollutants can be rapidly oxidized and decomposed to remove odor [41]. When the exhaust gas enters the microwave photolysis module, the microwave ultraviolet lamp in the module emits high-energy ultraviolet light [42]. On the one hand, the oxygen molecules in the air are oxidized and cracked to produce O_3_. On the other hand, the chemical bond of the harmful gas is broken to form free atoms or groups. At the same time, O_3_ also participates in the reaction process, so that the harmful gas is finally cracked and oxidized to form simple and stable compounds, such as CO_2_ and H_2_O.

#### 2.4.4. Ozone Removal Module

**Figure 5:**
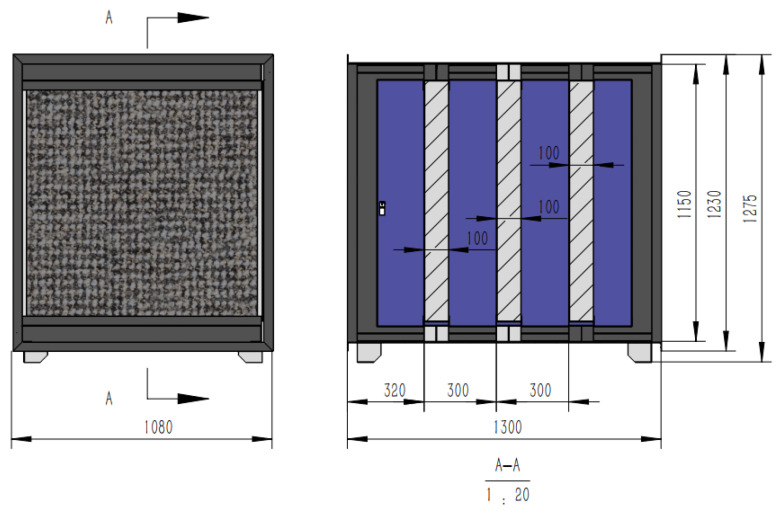
Ozone removal module.

Both the dielectric barrier discharge module and microwave photolysis module produce ozone molecules during the decomposition of noxious gases [43]. O_3_ is a potent oxidizing agent that aids in the elimination of harmful gas constituents. However, excessive concentrations of O_3_ can negatively impact the physical health of farm workers and pigs. The treated gas undergoes O_3_ elimination via an ozone removal module prior to discharge or return, which breaks down the ozone molecules generated by the dielectric barrier discharge module and microwave photolysis module to minimize the O_3_ concentration in the gas. The ozone removal module mainly consists of three layers, each of which is equipped with a large number of Mn-based catalysts [44], which can accelerate the decomposition of ozone molecules at standard ambient temperature, as shown in Table 3 and Figure 5.

## 3. Results

### 3.1. Animal Performance

Low-temperature plasma can result in the generation of a substantial quantity of potent oxidizing substances, with particular attention drawn to the production of low concentration O_3_, which may pose a certain level of risk to both pigs and farm workers [45]. Throughout the experiment, farm workers conducted irregular inspections of the pigs within the experimental premises and did not observe any evident abnormal behavior. The monitoring data revealed that the concentration of harmful gases remained within the safe range over a period of 60 days. On 7 February 2023, the survival rate of pigs for both House 1 and House 2 were recorded as 97% and 98.5%, respectively. This observation indicates that the operation of the deodorization equipment, which generates strong oxides, noise, and moving airflow, did not exert a significant impact on the pigs. Furthermore, the air purification effect achieved surpassed that of natural ventilation in a noteworthy manner.

### 3.2. Air Temperature and Relative Humidity

An optimal temperature is fundamental to ensure the normal functioning of biological activities within pigs, while air humidity has a direct impact on individual pigs’ ability to regulate their external environment. The effects of humidity fluctuations within pig facilities are contingent upon temperature, and the combined influence of humidity and temperature affects the body’s ability to dissipate heat. This study was conducted during the local autumn and winter seasons, characterized by a dry atmosphere and lower indoor temperatures. The outdoor temperature and humidity exhibited substantial fluctuations, whereas the indoor humidity was consistently controlled at a relatively stable level.

**Figure 6:**
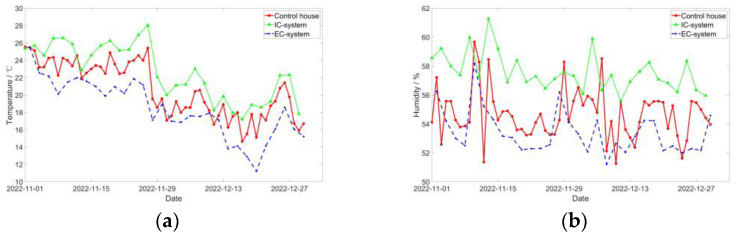
Temperature and relative humidity trend: (**a**) temperature and (**b**) relative humidity.

Figure 6 and Table 4 illustrate that the average temperature in the IC-system was significantly higher compared to the control group, whereas the average temperature was lowest under the EC-system. The average relative humidity in the IC-system consistently remained at a high level, followed by the control group, and the EC-system demonstrated the lowest humidity levels. The IC-system remained in a closed state, devoid of any air exchange with the outside, resulting in the maintenance of relatively stable and elevated temperature and humidity levels. Conversely, the control house, House 2, and the EC-system experienced air exchange, facilitated by fans that enhanced indoor airflow, creating a stronger negative pressure. Consequently, in the EC-system, the pig house admitted a greater influx of outdoor air, leading to higher temperature and humidity levels within the house compared to the control house.

### 3.3. NH_3_ and H_2_S

Both NH_3_ and H_2_S are highly toxic gases that pose significant risks to pigs. Prolonged exposure to low concentrations of these gases weakens the physical well-being of pigs, rendering them more susceptible to certain diseases [46]. Consequently, their feed intake, daily weight gain, and reproductive capacity decline. Excessively high concentrations directly induce noticeable pathological reactions and symptoms in the pig herd.

**Figure 7:**
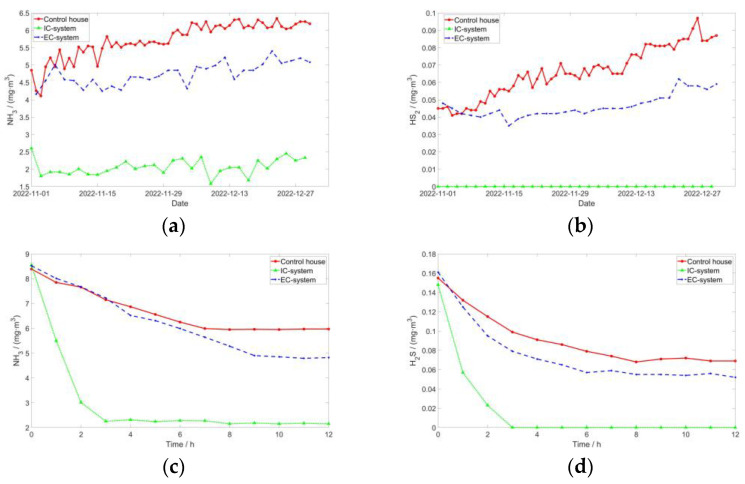
Trends in NH_3_ and H_2_S changes: (**a**) NH_3_ per day during the experimental period; (**b**) H_2_S per day during the experimental period; (**c**) NH_3_ measurements on 20 December; and (**d**) H_2_S measurements on 20 December.

Figure 7 and Table 4 illustrate that, irrespective of the operating mode, House 1 exhibited significantly reduced levels of NH_3_ and H_2_S compared to the control house. Specifically, the EC-system witnessed a decrease of 17.1% and 28.57% in NH_3_ and H_2_S concentrations, respectively, while the IC-system experienced a reduction of 63.87% and 100% (not detected), respectively. Through the scrutiny of alterations in gas concentrations on 20 December, it was ascertained that the IC-system expeditiously diminished NH_3_ and H_2_S to their nadirs within a span of 3 h. In contrast, the control house and EC-system exhibited a gradual descent, with the latter achieving a relatively modest minimum value. There were significant differences between the IC-system, EC-system, and control house. The closed-loop ventilation mechanism of the IC-system, which remains unaffected by outdoor conditions, offers the most accurate representation of the equipment’s deodorization efficiency.

The results indicate that this deodorization device demonstrates the highest efficacy in removing NH_3_ and H_2_S when compared to air exchange with the outdoors. The EC-system, characterized by increased ventilation, exhibited lower NH_3_ and H_2_S content than the control house. However, the presence of other pig houses or biogas digesters within the pig farm environment leads to a certain level of NH_3_ and H_2_S in the atmosphere, thereby diminishing the deodorization capabilities of the EC-system relative to the IC-system.

### 3.4. CO_2_

The CO_2_ present within pig houses primarily originates from the respiration of the pigs, with emissions increasing in line with their weight. Excessive CO_2_ levels within the house can induce chronic hypoxia in the pigs, leading to restlessness [47]. Prolonged exposure to hypoxia can result in reductions in both feed intake and daily weight gain in the pigs.

**Figure 8:**
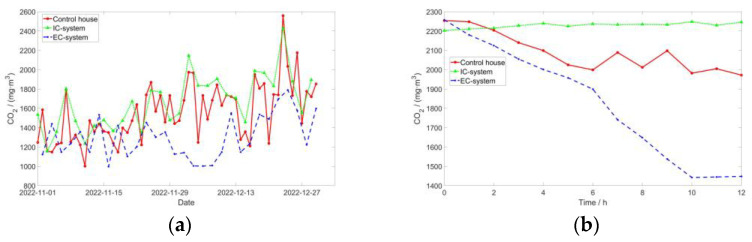
Trends in CO_2_ changes: (**a**) CO_2_ per day during the experimental period and (**b**) CO_2_ measurements on 20 December.

Figure 8 and Table 4 demonstrate that the IC-system exhibits the highest CO_2_ concentration, while the EC-system displays the lowest levels, in accordance with the GB/T17824.3-2008 standard. In comparison to the control house, the CO_2_ concentration increased by 7.01% in the IC-system and decreased by 16.43% in the EC-system. On 20 December, through scrutiny of the fluctuations in the CO_2_ concentration, it was discerned that the EC-system yielded optimal efficacy in diminishing CO_2_ levels, attaining its nadir at the tenth hour. Conversely, the control house exhibited a more gradual decline, whereas the IC-system, ensconced within a sealed environment, sustained a comparatively elevated CO_2_ concentration. There were significant differences between the EC-system and the IC-system, as well as with the control house, while there was no significant difference between the IC-system and the control house. This suggests that the plasma deodorization device does not have a pronounced effect on reducing CO_2_ levels, with ventilation remaining the primary method for CO_2_ control.

### 3.5. O_3_

During the operation of the plasma equipment, the production of O_3_ occurred, and its high concentration could have had implications for the physical health of both pigs and farm workers [48]. However, it is important to note that O_3_ is inherently unstable under room temperature and pressure, with it undergoing simultaneous generation and decomposition. Numerous ozone molecules were consumed through chemical reactions with harmful gases. When the equipment was in operation, the airflow within the ventilation duct attained a high-speed state, with it swiftly passing through the ozone removal module, thereby limiting the treatment efficiency. As a result, some O_3_ might still have found its way back into the pig house. Table 4 shows that no detectable levels of O_3_ were observed in all three sets of data, thus meeting the standard requirements of GB/T1883-2002.

## 4. Discussion

Table 4 presents the air quality monitoring data obtained from two pig houses, specifically focusing on House 1 divided into IC and EC modes. A comparative analysis of the three datasets was conducted to determine their differences. The results revealed that, the average temperature and average relative humidity of the EC-system were only 2.23 °C and 3.06% lower, respectively, than those of the control house, House 2, over a 60-day period. However, the average concentration of CO_2_ exhibited a 16.43% decrease. All air indicators, with the exception of relative humidity, exhibited significant differences. In comparison to the control house, the IC-system demonstrated a significant reduction in the concentrations of NH_3_ and H_2_S, namely 63.87% and almost 100%, respectively, with extremely significant differences observed. The IC-system experienced an increase in average temperature, average relative humidity, and CO_2_ levels of 2.04 °C, 2.93%, and 7.01%, respectively. When comparing the monitoring data between the IC-system and the EC-system, it was evident that all indicators exhibited significant differences.

These findings indicate that the device, through the combined operation of the DBD module, the microwave photolysis module, and the ozone removal module, effectively removes NH_3_ and H_2_S. The indoor temperature, relative humidity, and CO_2_ concentration may be more closely related to the ventilation volume within the pig houses. No matter how the deodorization device operates, the indoor O_3_ concentration always meets the relevant safety standards. Ventilation increased the actual air exchange rate within the houses and effectively reduced the CO_2_ levels, but its impact on water-soluble NH_3_ and H_2_S was limited. Therefore, the EC-system was better suited to reduce the CO_2_ concentration, whereas the IC-system was more effective in reducing the NH_3_ and H_2_S concentrations. Moreover, switching between different working modes can assist in adjusting the indoor temperature and humidity levels.

**Figure 9:**
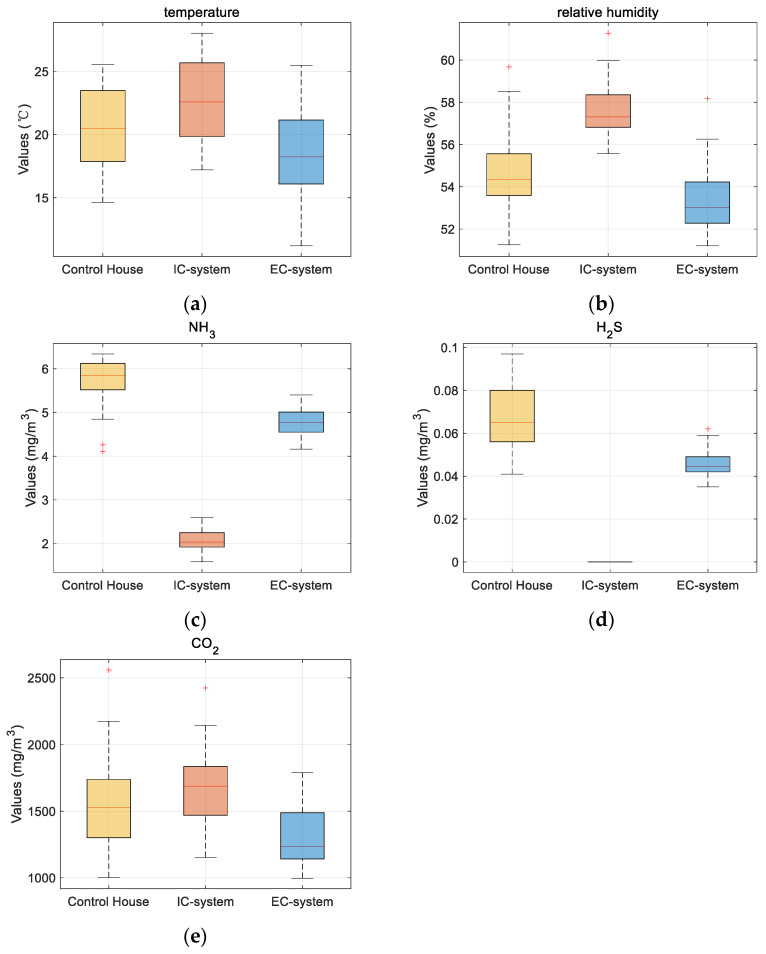
Changes in the monitoring indicators of the control house and the IC/EC-system: (**a**) temperature; (**b**) relative humidity; (**c**) NH_3_; (**d**) H_2_S; and (**e**) CO_2_.

From Figure 9a,b, it is discernible that the temperature and humidity levels within the IC-system surged markedly in contrast with the control house. Conversely, the EC-system exerts a counteracting influence. Furthermore, the median positions of data clusters in both graphs manifest in similarity, implying a marginal impact on their data dispersion. Notably, Figure 9c,d show that both the IC-system and EC-system exhibit efficacy in attenuating the indoor concentrations of NH_3_ and H_2_S, with the IC-system demonstrating a pronounced effect. The data distribution for the EC-system appears more concentrated, indicative of a challenge in further diminishing NH_3_ and H_2_S concentrations beyond a certain threshold. In Figure 9c, it is evident that the EC-system remains the primary modality for reducing the CO_2_ concentration, with the median line (red line) notably lower, denoting enhanced efficacy in reducing the CO_2_ concentration under most circumstances.

Furthermore, Figure 9 exhibits isolated instances of aberrant data (red plus sign), attributed primarily to two factors. Firstly, the indoor milieu was susceptible to the impact of external conditions, with notable winter climate fluctuations and sporadic instances of extreme weather. Secondly, the austere conditions within the pig house, coupled with the deodorization apparatus and data sensors, may contribute to the accumulation of dust and other phenomena. In the absence of timely cleaning, these circumstances may result in data inaccuracies.

## 5. Conclusions

The findings of this study revealed a substantial reduction in the concentration of NH_3_ and H_2_S within House 1 over the course of the two-month experimental period. Among the different modes tested, the IC-system demonstrated the most notable effect, with in exhibiting superior insulation and moisturizing properties as well. However, the EC-system proved more effective in diluting CO_2_. Consequently, the plasma deodorization device exhibited a discernible improvement in air quality within the house and exhibited remarkable sensitivity to the concentrations of NH_3_ and H_2_S. The levels of CO_2_ and O_3_ generated in the IC-system also remained within the safe range. The presence of the external circulation mode offers a viable solution for further reducing CO_2_ levels. In practical application, internal circulation serves as the primary ventilation method, while periodically switching to external circulation to expel excess carbon dioxide and ozone emerges as the optimal operational mode.

In this paper, low-temperature plasma technology harnessed greater energy generation capabilities via dielectric barrier discharge. When integrated with microwave photolysis technology, it had the ability to disintegrate gaseous pollutants into non-toxic substances, thereby efficiently diminishing the concentration of polluted gases. It was equipped with an ozone removal module to ensure that all monitoring indicators remained within the confines of health and safety regulations. Simultaneously, this equipment exhibited remarkable processing efficiency by rapidly decomposing harmful substances within an exceedingly short duration of contact. It facilitated round-the-clock remote monitoring and control, streamlined manual operations, and effectively reduced labor costs.

Nevertheless, the apparatus expounded upon in this discourse grapples with challenges encompassing an extensive spatial requirement, limited coverage scope, and elevated expenditures. Moreover, the arduous conditions within the pig house led to the accretion of dust on the purification apparatus and monitoring sensors, thereby compromising their operational efficacy and mandating regular manual upkeep. In subsequent research, our focus will pivot towards achieving miniaturization, augmenting efficiency, and curbing expenses to address the aforementioned impediments. Additionally, we envisage undertaking trials in a more eclectic array of environments to substantiate the resilience of the system.

## Figures and Tables

**Table 1:** Parameters of the different models of fans.

Fan Model	Blade Diameter (mm)	Blade Speed (r/min)	Motor Speed (r/min)	Air Volume (m^3^/h)	Power (W)
JDXS-710	710	560	1350	17,000	370
JDXS-900	1400	560	1350	24,000	550
JDXS-1000	1000	600	1400	28,000	750
JDXS-1250	1250	439	1400	38,000	1100

**Table 2:** Odor removal device parameters.

Module		Parameters	
Treatment capacity	10,000 m^3^/h	Size (mm)	5565 × 1335 × 3000
Filter plate	Activated carbon sponge	Size (mm)	1000 × 1000 × 50
Electric field	201 stainless steel	Size (mm)	780 × 485 × 290
	2 installation positions	Voltage/power	220 V/300 W
DBD module	6 installation positions	Voltage/power	220 V/50 W
Ultraviolet lamp	6 installation positions	Voltage/power	220 V/300 W
Photocatalyst	Aperture 4 mm	Size (mm)	1000 × 1000 × 3
Catalyzer	Four groups	Size (mm)	1000 × 1000 × 100
Induced draft fan	Axial-flow type	Power	5.5 kW
Main body	The frame was welded with 40 × 40 angle iron	Material	235 Q

**Table 3:** Catalyst parameters.

	Specifications
Shape	Cylindrical particles
Particle size (mm)	Φ (2–4) × (5–15)
Density (kg/m^3^)	700–800
Model	DXO3-1
Function	Catalytic decomposition of O_3_ and oxidation of VOCs
Component	Mn-based catalysts
Intake air humidity	0–98% (RH)
Intake air temperature	Ordinary temperature

**Table 4:** Indoor air quality indicators.

	Control House Value	EC-System		IC-System		Sig^c^
		Value	Sig^a^	Value	Sig^b^	
Temperature (°C)	20.64	18.41	<0.05	22.68	>0.05	<0.05
Relative humidity (%)	54.65	51.59	>0.05	57.58	>0.05	<0.05
NH_3_ (mg/m^3^)	5.73	4.75	<0.05	2.07	<0.01	<0.05
H_2_S (mg/m^3^)	0.07	0.05	<0.05	/	/	/
CO_2_ (mg/m^3^)	1559.03	1302.93	<0.05	1668.37	>0.05	<0.01

Sig^a^ represents the differences between the EC-system and the control house; Sig^b^ represents the differences between the IC-system and the control house; Sig^c^ represents the differences between the IC-system and the EC-system.

## Data Availability

The data presented in this study are available on request from the corresponding author.
